# Structural Anomalies Detection from Electrocardiogram (ECG) with Spectrogram and Handcrafted Features

**DOI:** 10.3390/s22072467

**Published:** 2022-03-23

**Authors:** Hongzu Li, Pierre Boulanger

**Affiliations:** Department of Computer Science, Faculty of Science, University of Alberta, 116 St and 85 Ave, Edmonton, AB T6G 2R3, Canada; pierreb@ualberta.ca

**Keywords:** electrocardiogram, deep learning, signal processing, machine learning, anomaly detection

## Abstract

Cardiovascular diseases are the leading cause of death globally, causing nearly 17.9 million deaths per year. Therefore, early detection and treatment are critical to help improve this situation. Many manufacturers have developed products to monitor patients’ heart conditions as they perform their daily activities. However, very few can diagnose complex heart anomalies beyond detecting rhythm fluctuation. This paper proposes a new method that combines a Short-Time Fourier Transform (STFT) spectrogram of the ECG signal with handcrafted features to detect heart anomalies beyond commercial product capabilities. Using the proposed Convolutional Neural Network, the algorithm can detect 16 different rhythm anomalies with an accuracy of 99.79% with 0.15% false-alarm rate and 99.74% sensitivity. Additionally, the same algorithm can also detect 13 heartbeat anomalies with 99.18% accuracy with 0.45% false-alarm rate and 98.80% sensitivity.

## 1. Introduction

Cardiovascular diseases (CVD) are the leading cause of death worldwide, with nearly 17.9 million deaths per year. CVD continues its decades-long rise in low-income and middle-income countries. Even in high-income countries with accessible healthcare systems, the age-standardized rate of CVD has begun to rise [1]. Therefore, there is an urgent need to focus on implementing cost-effective policies and technological solutions to improve this situation by emphasizing early CVD detection, which can be life-saving, especially in younger populations [2]. This is even more true in many low-income and middle-income countries because of limited access to medical equipment and the high cost of medical services. To help solve this situation, the detection and treatment using low-cost heart sensors and new algorithms that automatically detect heart anomalies are essential.

The electrocardiogram (ECG) is the most commonly used tool to detect heart conditions. It is a quick, safe, and painless way for physicians to check their heart rate and rhythm and detect potential heart disease signs. Standard ECG practice consists of installing the electrodes on the patient at rest and then measuring the lead’s voltages for 10 s. Next, a cardiologist reads the results and performs a diagnostic. The problem with this approach is that the patient needs to go to a clinic to be measured and hope that the cardiologist can detect abnormal heart activities during the visit. This is far from what is necessary for the early detection of CVD. Ideally, continuous 24/7 ambulatory ECG monitoring devices are needed to measure heart activities during normal daily activities or at night. These ambulatory ECG monitoring devices are connected wirelessly to a secure central cloud service where data recordings are processed to detect heart anomalies. The device must be low-cost, capable of dealing with motion artifacts and network limitations. At the cloud, an algorithm must automatically reduce motion artifacts from these long-duration remote recordings and then identify the anomalies that a cardiologist will confirm. A novel algorithm for the reduction of motion artifacts was presented in a previous paper by the authors [3]. This paper deals with the detection of heart anomalies on long recordings using a neural network using handcrafted input features extracted from the ECG spectrogram.

**Figure 1:**
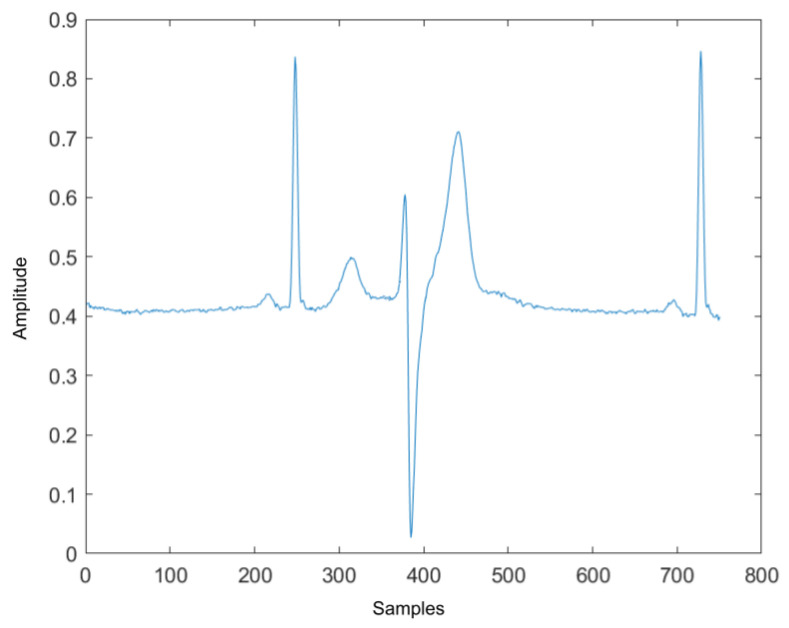
Normal rhythm contains abnormal heartbeat.

Anomaly detection on ECG signals can be divided into rhythm and heartbeat classifications. Rhythm classification focuses on finding abnormal rhythms among normal rhythms. To find a rhythm anomaly, one must process multiple heartbeats. Moreover, the heartbeat classification focuses on finding the pattern of one heartbeat signal [4]. Therefore, the proposed algorithm must first process the entire ECG record and then detect abnormal rhythms (Figure 1).

In Section 1.1, the state-of-the-art of rhythm and heartbeat classification algorithms are reviewed. Next, Section 2 will describe the proposed algorithm where ECG signals are transformed into a Short-Time Fourier Transform (STFT) spectrum followed by handcrafted features to classify ECG anomalies using a convolutional neural network architecture. Section 3 describes the experimental setup and compares the detection results to state-of-the-art algorithms. The explanation of the advantages of this new algorithm is concluded in Section 4.

### 1.1. Related Work

As stated earlier, anomaly detection consists of two parts: rhythm and heartbeat classifications. Rhythm classification determines the type of the rhythm anomalies such as Normal Sinus Rhythm (NSR), Atrial Fibrillation (AF), Ventricular Flutter (VF), and so on. The ECG segments for rhythm classification are usually contained in several heartbeats, which could be normal or abnormal. Heartbeat classification takes an ECG signal that only includes one heartbeat as the input and outputs the heartbeat type such as normal heartbeat, Left/Right bundle branch block beats, premature beats, etc. The following sections introduce and explain the literature on rhythm and heartbeat classifications.

The goal of rhythm and heartbeat classification is to find abnormal rhythms and heartbeats on a continuous ECG signal. The difference is that the input for rhythm classification needs to contain several heartbeats, and the input for heartbeat classification only has one heartbeat. However, they share a similarity in that they consist of two main parts: feature extraction and classification. In the feature extraction part, many researchers extract the features that represent the ECG signal. In the classification part, models are trained with the extracted feature and then classified.

#### 1.1.1. Feature Extraction

According to the literature, there are two main conventional features: derived and morphological [4]. The derived features are calculated from the ECG signal using feature detectors. The common derived features found in the literature are: Auto-Regressive (AR) coefficients [5]; Discrete Wavelet Transform (DWT) coefficients [6,7]; Eigen Vectors [8]; Dual-Tree Complex Wavelet-Transform coefficients [9]; Intrinsic Mode Functions from Empirical Mode Decomposition [10]. The other morphological features deal with the morphological information found in the ECG signal. The most used morphological features found in the literature are: QRS complex duration and peak values; P-wave duration and peak value; T-wave duration and peak value; PR interval length; QT interval length and so on [11,12]. In [4], one can see a more detailed list of the most common morphological features used for ECG signal processing. There are some advantages and drawbacks to both types of features. The derived features usually represent the ECG signal in the frequency domain. Therefore, the noisy components in the ECG signal can be easily discarded during the process even though this means that some essential information in the time domain may be lost, such as peak values, interval duration, etc. Moreover, the derived features may not distinguish between different rhythms or heartbeats. Morphological features, on the other hand, focus on the time domain even though they are easily contaminated by noise. They also represent the same information that Cardiologists use to interpret ECG recordings. Other than the standard derived and morphological features, the spectrogram of the ECG signal was proposed by [13,14] for rhythm and heartbeat classification. Compared to the standard features, a spectrogram contains information from both the time and frequency domains. The drawback is that the spectrogram is a 2D image. Therefore, traditional 1D classification algorithms cannot be applied.

#### 1.1.2. Classification Tool

Once the feature vectors are extracted from the ECG signal; a classification algorithm is needed to classify each condition. The Euclidean distance is one of the most popular metrics to compare two vectors’ similarities. It computes the distance between two same-length vectors, and one can cluster the input vectors based on the computed distance. The paper by [15] shows an example of using Euclidean distance for anomaly heartbeat detection. K-means clustering is the most common clustering method that uses Euclidean distance to cluster data entries to different clusters. In [16], the K-means clustering method is used to classify heartbeat into normal and abnormal classes. The Euclidean distance and its corresponding clustering method are fast and simple methods to distinguish different classes. However, it has a high requirement on the extract features. The difference between feature vectors must be large to be assigned into different clusters. In addition, it is easily affected by data outliers and noise.

Another common classification tool is used by traditional machine learning classification algorithms. The algorithms build a mathematical model on collected training data to perform anomalies recognition. There are some popular algorithms found in the literature: Kth Nearest Neighbor [17], Linear Discriminant Analysis [18], Support Vector Machine [7,12], Multi-Layer Perceptron Neural Network [9]. These algorithms rely heavily on feature selection. Therefore the raw ECG signal could not be the input of such algorithms. Compared to the Euclidean distance, the training time is much longer for these machine learning algorithms, and because of the size of the training dataset, a small number of outliers in the dataset will not affect the trained model.

With the rapid development of fast and low-cost computing hardware, deep learning neural networks (DNN) can be used to reduce the need to extract hand-crafted features from an ECG signal. In many applications, they can generate more accurate classifiers. However, DNN often requires a massive amount of data for training. Convolutional Neural Network (CNN) [19] model is one of the most popular artificial neural network. It is mostly used for images and signals analysis and segmentation. The most important layer in the CNN is the convolution layer. The convolution layer can reduce the input so that the network can process the local regions instead of the whole input [20]. Therefore, one advantage of CNN is that it could take a raw ECG or the spectrogram image as the input without the need for feature extraction. Another advantage of the CNN is that it may not require the preprocessing step since the convolution layer can eliminate the unwanted noise during training. There are several researchers who have explored the potential of CNN for rhythm and heartbeat classification [13,21,22,23].

Recurrent Neural Network (RNN) is another popular deep learning neural network class used to classify ECG. This neural network is specialized in processing sequential data such as time series, text and so on. Compared to feed-forward neural networks such as CNN, RNN uses past features that affect the present such as the well-known long short-term memory (LSTM) networks [24]. RNN could keep the useful information of the input and discard the rest from the beginning of the training. Therefore, while processing ECG signals, the prediction of the previous rhythms or heartbeats will also affect the decision of the current one. Both RNN and LSTM networks have been proven to be effective for rhythm, and heartbeat classification by several researchers such as [8,25].

## 2. Materials and Methods

The following section describe the public ECG dataset used to test and train the proposed algorithm (see Table 1 and Table 2 third columns).

### 2.1. Data Description

The test ECG datasets are from the MIT-BIH database [26] and the European ST-T database [27] both located in the PhysioNet database [28]. The MIT-BIH data contains 48 half-hour excerpts of two-channel ambulatory ECG recordings obtained from 47 subjects. The data is fully annotated with 15 different rhythm classifications. The European ST-T database consists of 92 annotated 2 h excerpts of two-channel ambulatory ECG recordings from 79 subjects with 10 different rhythm classifications, and a complete description of the rhythms is shown in Table 1. similarly, a complete description of the heartbeat is shown in Table 2. In these tables, M is short for the MIT-BIH database, and E is short for the European ST-T Database.

### 2.2. Methodology

As described previously, anomaly detection can be divided into abnormal rhythm detection and abnormal heartbeat detection. First, abnormal rhythm detection will find the abnormal rhythms in the ECG record and label them. However, some rhythms may be labeled as normal rhythm, they could contain abnormal heartbeat (an example can be seen in Figure 1). Therefore, the second part of the algorithm will go through all heartbeats in the normal rhythm and label the irregular heartbeats. This section describes the steps of the algorithm and the features used to model the rhythms and heartbeat signal.

#### 2.2.1. Signal Pre-Processing

The signal pre-processing steps consist of re-sampling, normalization, data extraction, and balancing. The ECG signals in the MIH-BIH arrhythmia dataset have a 360 Hz sampling frequency, and the signals in the European ST-T dataset have a 250 Hz sampling frequency. To make the sampling frequency at the same rate, both signals were down-sampled at a rate of 250 Hz, with an antialiasing lowpass Filter provided by Matlab [29]. The algorithm starts by inserting 250 zeros to the original signal $S{\left(t\right)}_{orig}$ to obtain the up-sampled signal $S{\left(t\right)}_{up}$. Then $S{\left(t\right)}_{up}$ is filtered by an FIR antialiasing filter that uses a Kaiser window method to maintain the original shape of $S{\left(t\right)}_{orig}$. Finally, 250 samples in the $S{\left(t\right)}_{up}$ are discarded to obtain the down-sampled signal $S{\left(t\right)}_{down}$. Next, the signals S(t) from both datasets are normalized between 0 and 1 using the min–max normalization formula:

(1) $$S\left(t\right)=\frac{value(t)-min}{max-min}.$$

#### 2.2.2. Rhythm and Heartbeat Signal Extraction

The ECG signal is split into 3-s segments using a sliding window method to extract the rhythm data. The window size is chosen to be 3× sample frequency, and the step size is chosen to be 1× sample frequency. The heartbeat data are extracted using the provided heartbeat annotation location. The R peak locations of the last heartbeat and the following R peak will be the beginning and ending points of the current heartbeat. After rhythm and heartbeat extraction, the extracted data is used to compute the spectrogram and features for data modeling.

#### 2.2.3. Spectrogram Using Short-Time Fourier Transform

Traditional signal-processing approaches assume that the signal is stationary, which is not true, especially for ECG. Hence, time or frequency descriptions alone are insufficient to provide a comprehensive analysis of the ECG signals [30]. Similar to speech analysis, time-frequency analysis is much more suitable for nonstationary signals. Using Short-Time Fourier Transform (STFT), one can determine the frequency and phase content of local sections of a signal as it changes over time [30]. STFT is a sequence of Fourier transforms of a windowed signal. The STFT matrix is the combination of the Discrete Fourier Transform (DFT) of each windowed segments which can be expressed as $X\left(f\right)=[X_{1}\left(f\right),X_{2}\left(f\right),X_{3}\left(f\right),\cdots ,X_{m}\left(f\right),\cdots ,X_{k}\left(f\right)]$ [31], where *k* is the number columns of the spectrogram.

**Figure 2:**
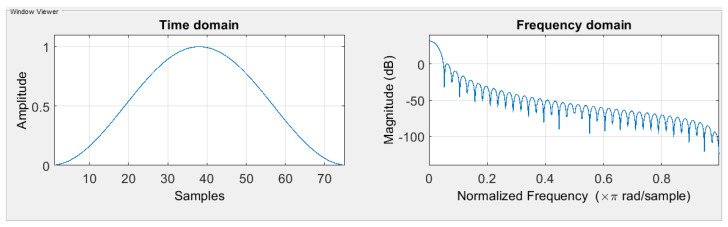
Hanning window function in the time domain and the frequency domain.

To compute the STFT spectrum, one must choose the window function and size. The window function selected must be adapted to the signal frequencies. The window size affects the amount of time and frequency information presented in the spectrogram. If the spectrogram is computed using a long window, then the spectrogram has more Discrete Fourier Transform (DFT) samples but less time resolution. On the other hand, if the spectrogram is computed using a short window, then the spectrogram has fewer DFT samples but better time resolution. The window size from 0.1 s to 1 s were tested to find a balance between frequency and time resolutions. In this search, based on many experiments, a window length *L* of 167 samples was chosen for the rhythm data, and 28 samples were chosen for the heartbeat data, which is 0.668 s and 0.11 s correspondingly. The best window function for the STFT is the Hanning window for both data (shown in Figure 2). The reason for selecting the Hanning window is that it ranged from 0 to 1 in amplitude, matching the normalized ECG signal range. Moreover, the shape of the Hanning window function is similar to the waves of the ECG signal.

**Figure 3:**
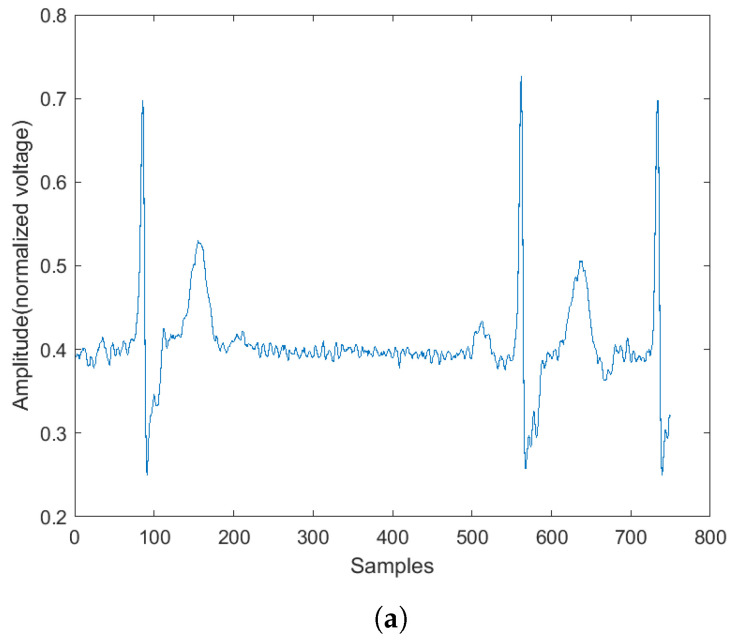

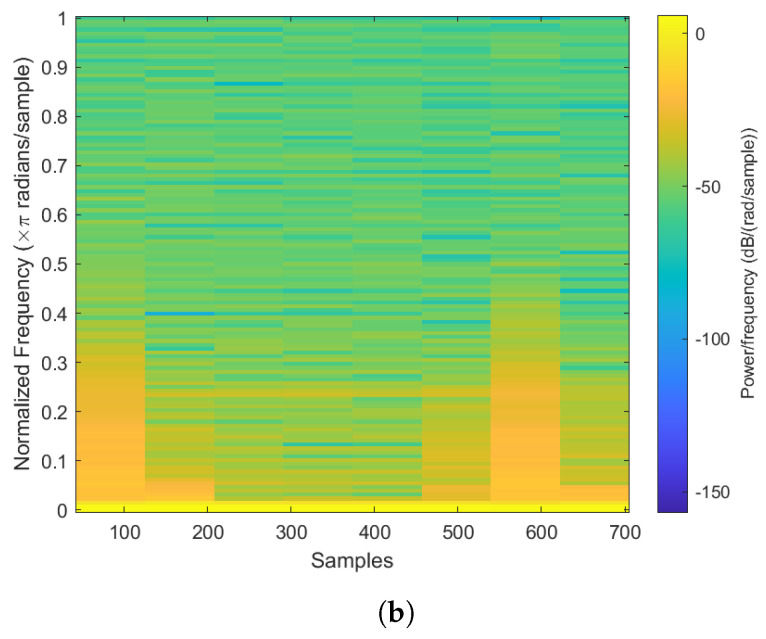
Abnormal ECG rhythm and its corresponding spectrogram. (**a**) Normal rhythm plot. (**b**) Normal rhythm spectrogram.

**Figure 4:**
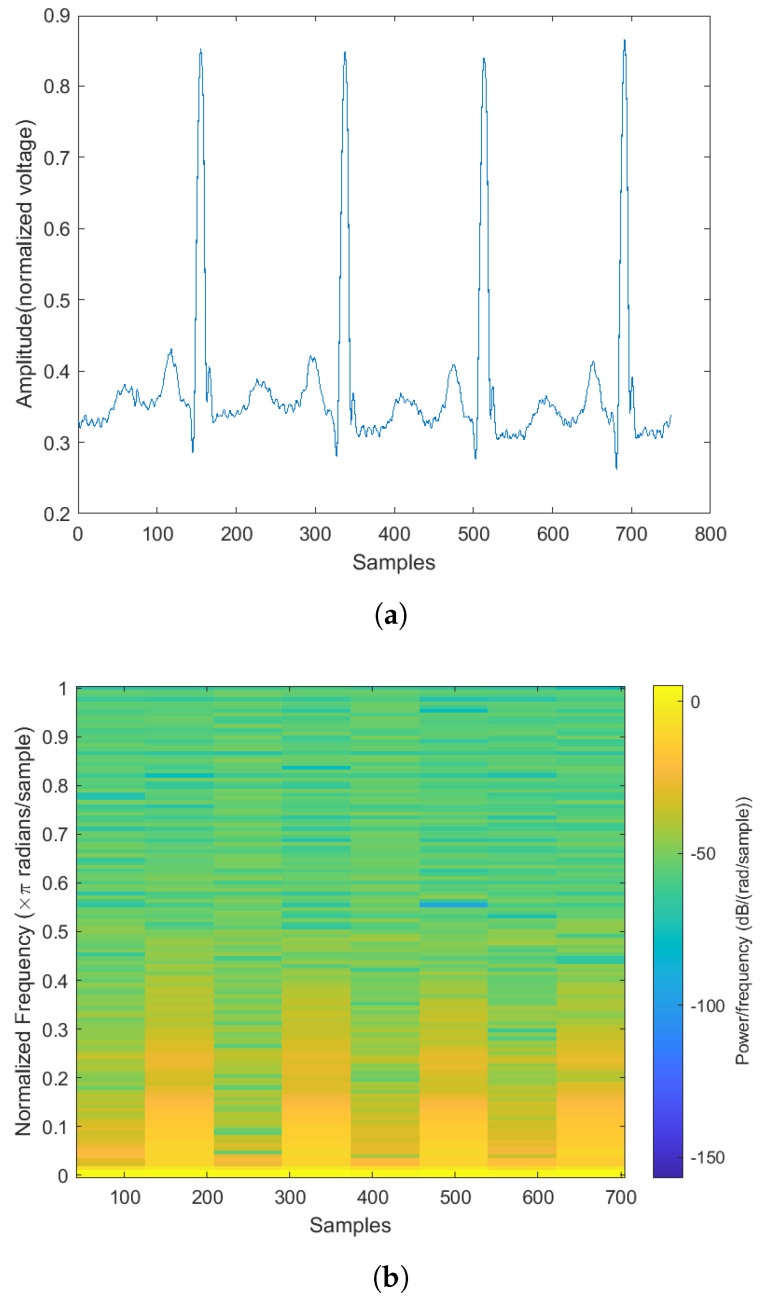
Normal ECG rhythm and its corresponding spectrogram. (**a**) Normal rhythm plot. (**b**) Normal rhythm spectrogram.

**Figure 5:**
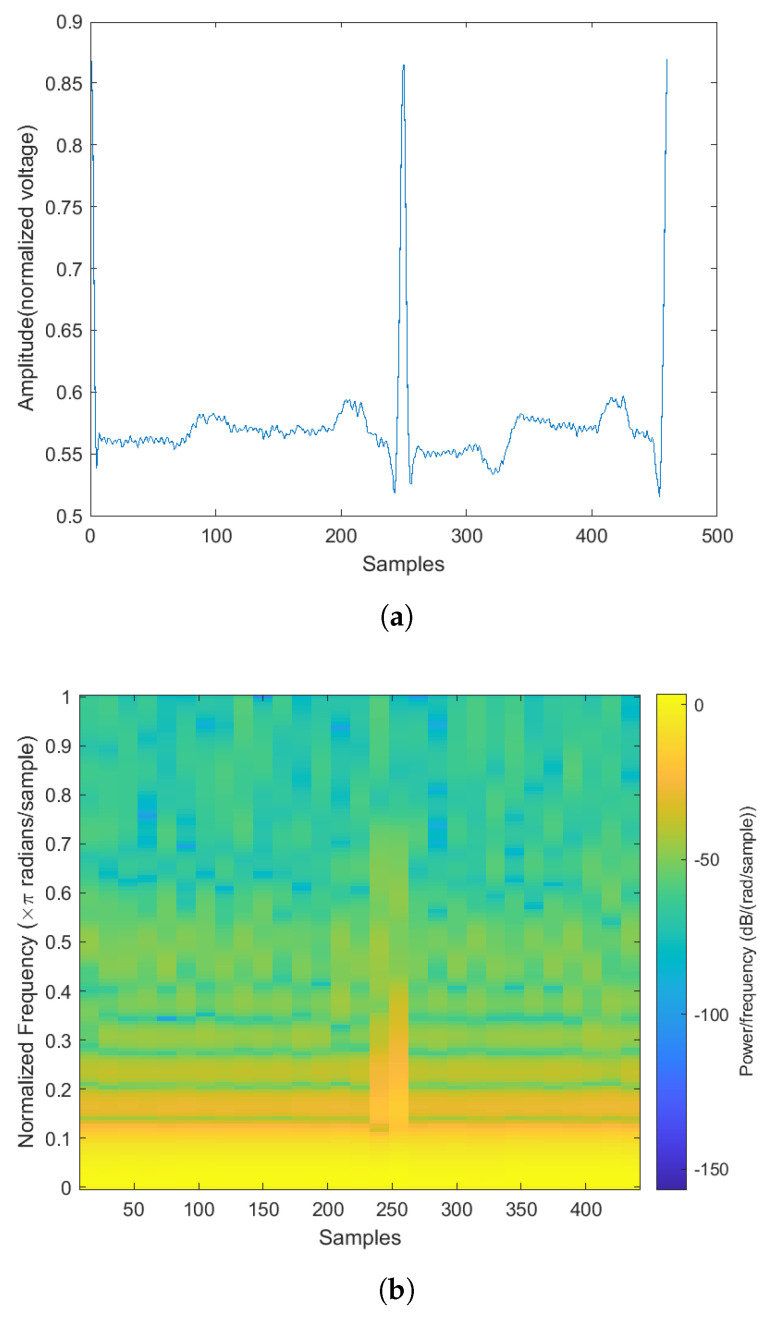
The normal heartbeat plot and spectrogram. (**a**) Normal heartbeat plot. (**b**) Normal heartbeat spectrogram.

**Figure 6:**
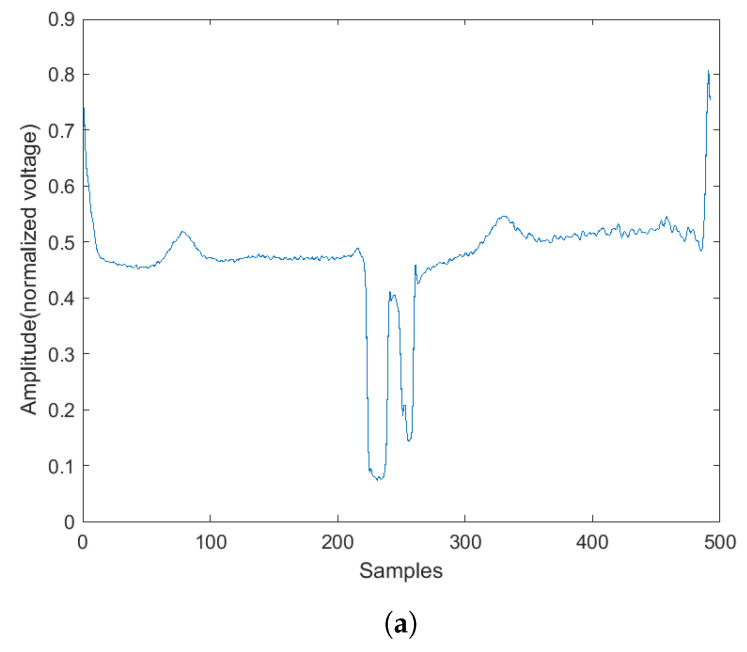

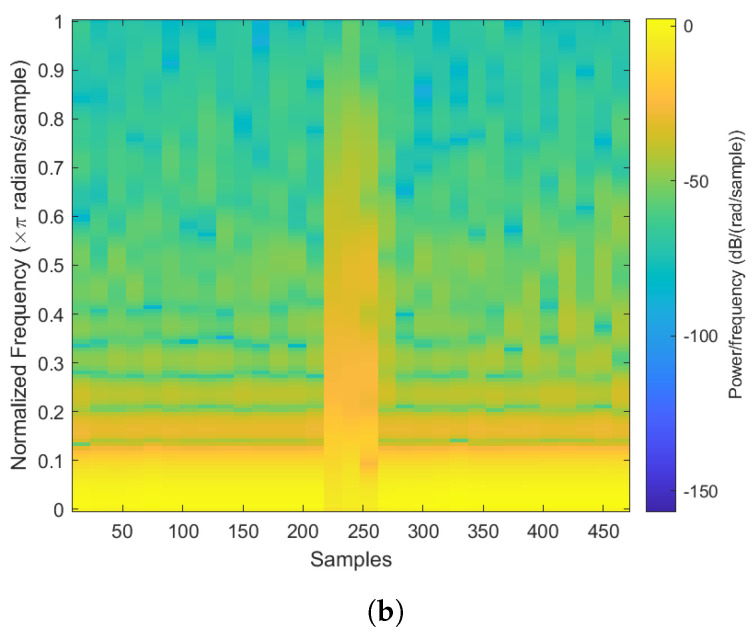
The abnormal heartbeat plot and spectrogram. (**a**) Abnormal heartbeat plot. (**b**) Abnormal heartbeat spectrogram.

Examples of the spectrogram and its corresponding ECG rhythm and heartbeat can be seen in Figure 3, Figure 4, Figure 5 and Figure 6. The y-axis for the ECG signal in the above figures were normalized voltage.

#### 2.2.4. Handcrafted Features Rhythm Classification

Sometimes, humans can quickly identify relationships between two objects where a computer cannot. In the proposed research, in addition to the extracted features from the Convolutional Neural Network, nine extra handcrafted features were added to improve the recognition performance. The feature vector for rhythm classification contains the number of R peaks detected from two algorithms, skewness, kurtosis, variance, average interval length, average QRS complex length, average PR interval length, average QT interval length. The features will be explained in the following sections.

#### 2.2.5. Detecting the Number of R Peaks

For this feature, two algorithms were used to detect the R peaks. One is the Pan–Tompkins algorithm [32], and the other one is the Shannon Energy Envelope Hilbert transform (SEEHT) [33]. The detected R peaks were included in the final feature vectors. The reason for using two R peaks detection algorithms is that they perform differently for different ECG signals. The Pan–Tompkins uses a filter bank that only keeps the R peaks on the ECG signal. The SEEHT algorithm calculates the Shannon energy envelope of each heartbeat on the ECG signal and then applies Hilbert transform to the Shannon energy signal. By finding the zero-crossing of the converted signal, one can detect the R peak locations. By comparison, the SEEHT has higher overall detection accuracy, and the Pan–Tompkins has higher abnormal heartbeat detection accuracy. For example, if one selects a record from the MIT-BIH database with 472 ventricular flutter (VF) beats, the Pan–Tompkins algorithm will detect 363 VF beats, and the SEEHT will only detect 177 VF beats. Therefore, by using both algorithms as features, one can improve detection accuracy.

#### 2.2.6. Statistics Features

Another handcrafted feature is a skewness measurement that computes the asymmetry of the ECG signal about its mean. Negative skewness indicates the signal is leaning right, and positive skewness indicates the signal is leaning left. The skewness estimate can be computed using the following equation:

(2) $$skewness=\frac{\mu^{3}}{\sigma^{3}},$$

where the μ is the mean, and the σ is the standard deviation.

The kurtosis describes the ‘peak’ of a signal. The kurtosis should be zero for a perfectly normal distribution. Signal with positive excess kurtosis indicates a high peak, and a signal with negative kurtosis indicates a flat-topped curve [34]. The kurtosis can be computed using the following equation:

(3) $$kurtosis=\frac{\mu^{4}}{\sigma^{4}},$$

where μ is the mean, and the σ is the standard deviation.

The variance of a signal computes how far the sample points are away from their mean. It can be computed using the following equation:

(4) $$Variance=\frac{1}{N-1}\sum_{i=1}^{N}|X_{i}{-\mu |}^{2},$$

where $X_{i}$ is the signal, *N* is the length of the signal, and μ is the mean of the signal.

#### 2.2.7. ECG Intervals Measurement

**Figure 7:**
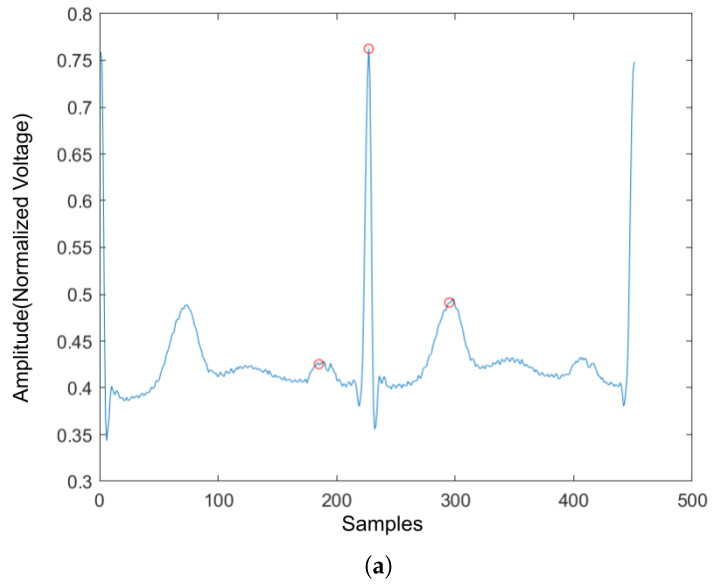

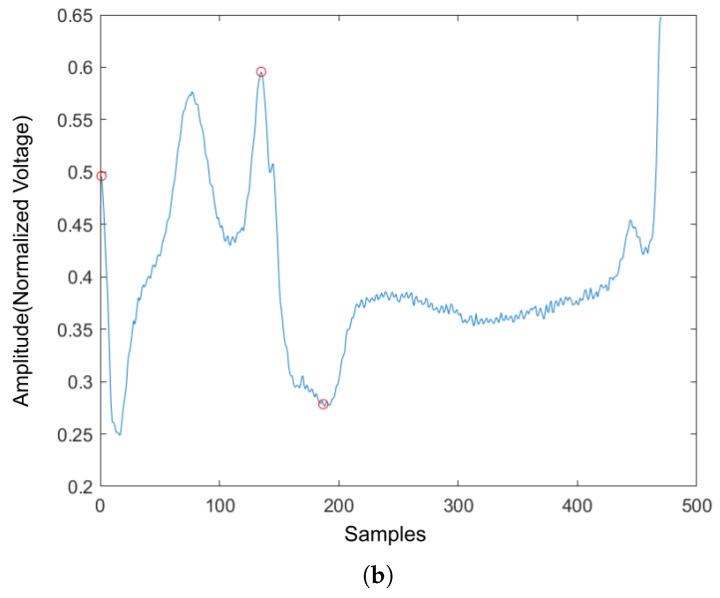
The P,R,T peak detection on both normal and abnormal heartbeat. (**a**) P,R,T peak detection on normal heartbeat. (**b**) P,R,T peak detection on abnormal heartbeat.

Four ECG interval measurements are also used as features. They are average RR interval length, average QRS complex length, average PR interval length, and average QT interval length. The detection tool used for detecting the Q wave and T wave was ‘ecgpuwave’, which is provided by PhysioNet [28]. The ‘ecgpuwave’ uses Pan–Tompkins algorithm [32] to detect the QRS complex. Then the detector detects the P peak and T peak based on the detected R peaks. For P and T peak detection, the detector defined search windows before and after the R peak. The detector searches the maximum and the minimum values in the defined window. The P and T wave peak is assumed to occur at the zero-crossing between the maximum and the minimum values in the search windows. Figure 7a,b show the examples of the P-QRS-T detection using the ‘ecgpuwave’ detector. These two examples represent the most common situation for detecting the P, R and T peaks on the heartbeats with the ‘ecgpuwave’ detector, which are the peaks on abnormal heartbeats that cannot be fully detected and the detection on normal heartbeats are perfectly detected. Therefore, for undetected peaks, its corresponding interval length and peak value will be set to 0.

#### 2.2.8. Handcrafted Features for Heartbeat Classification

The previous statistics features for the rhythm are also used for the heartbeat signal: skewness, kurtosis, and variance. However, there are still some features that are different from the rhythm. Since there is only one heartbeat in the heartbeat signal, the number of R peaks, average RR interval length, average QRS complex length, average PR interval length, and average QT interval length no longer exists. They are replaced with normalized R peak value, left RR interval length, right RR interval length, PR interval length, QRS complex length, QT interval length, In addition, as shown in Figure 7, the ’ecgpuwave’ detector could detect the peak location of the heartbeat signal, and the P wave and T wave peak value could also be retrieved. Therefore, the final two features added to the feature vector are normalized P wave peak and T wave peak values.

To sum up, there are a total of 11 features in the handcrafted feature vector which are normalized R peak value, left RR interval length, right RR interval length, skewness, kurtosis, variance, QRS complex length, PR interval length, QR interval length, normalized P wave peak value, and normalized T wave peak value.

#### 2.2.9. Network Architecture

**Figure 8:**
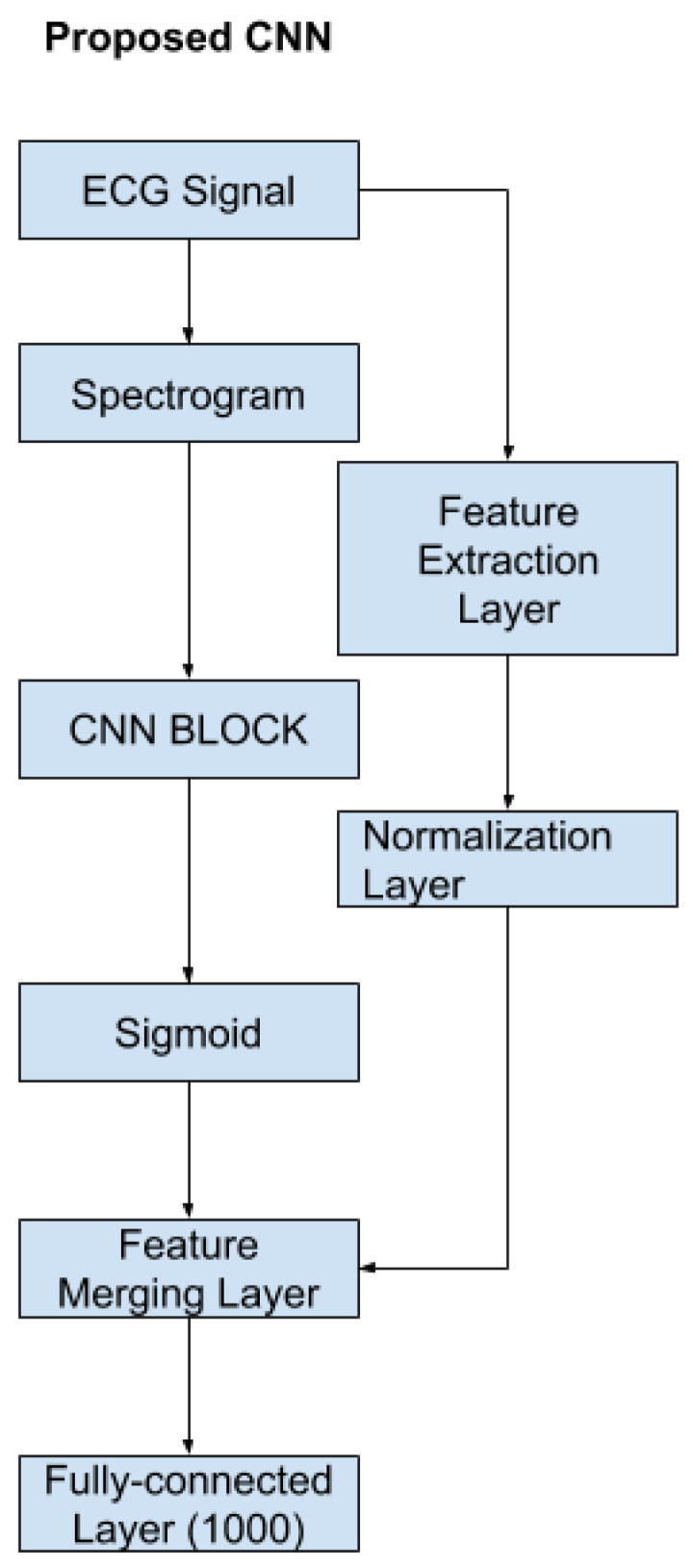
The proposed CNN.

**Figure 9:**
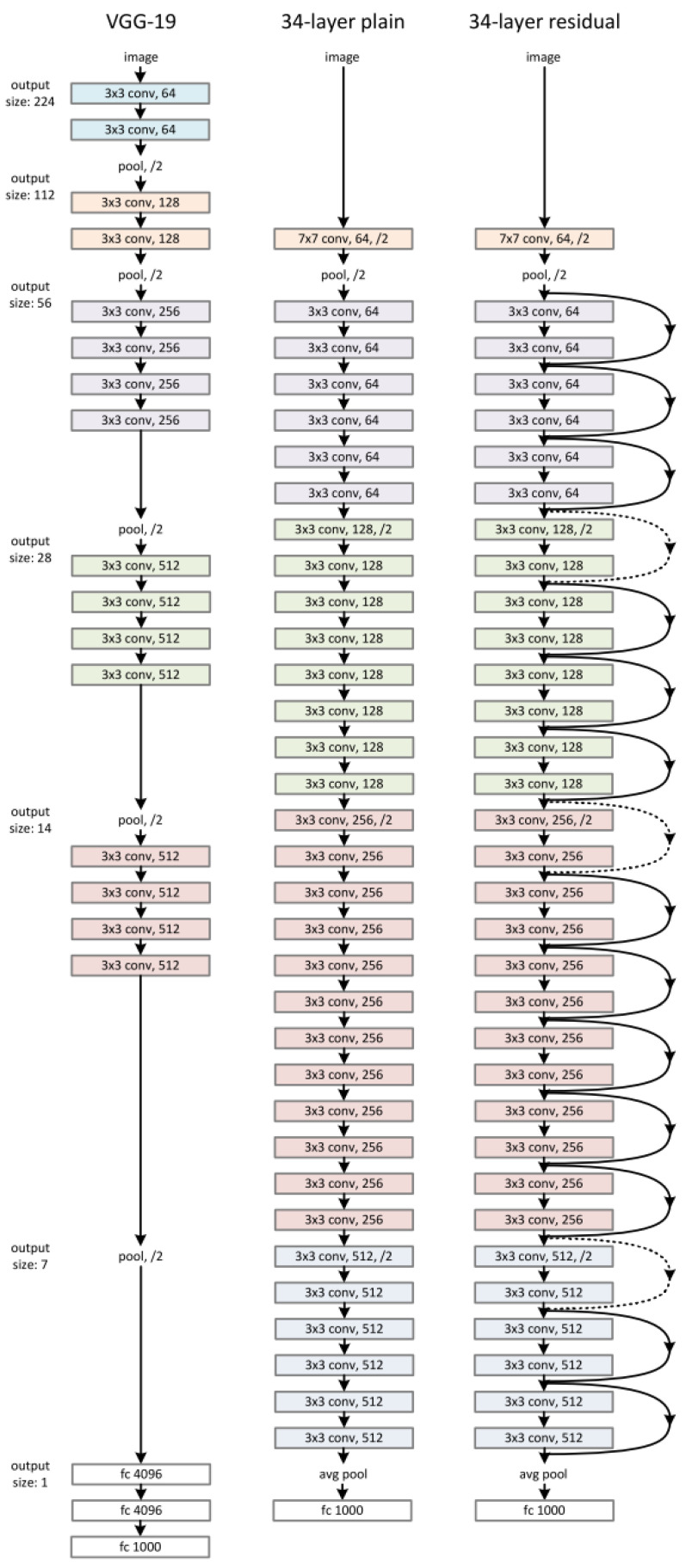
VGG-19 CNN, 34-layer plain CNN, 34-layer ResNet.

The proposed Convolutional Neural Network (CNN) consists of a feature-extraction layer, a normalization layer, a CNN block, and a feature-merging layer. The proposed CNN structure is shown in Figure 8. According to the figure, the original input is the raw ECG signal. From the original input, the program could produce the STFT spectrogram and the handcrafted features in Section 2.2.8. Once the handcrafted features are obtained, the normalization layer will normalize those features to a range between [0, 1] so that each feature contributes the same weight to the classification. The STFT spectrogram was sent to the CNN block and a sigmoid layer. The CNN block could be any popular CNN structure such as VGG-19 CNN, plain 34-layers CNN, 34-layers Residual Net (shown in Figure 9). The primary purpose of the CNN block was to extract the features from the spectrogram using convolutional layers. The sigmoid layer acts as a normalization layer, converting CNN features to be in the range [0, 1] so that the CNN features will have the same weight as the handcrafted features at the final layer. Once the CNN and handcrafted features are obtained, the feature-merging layer then combines the normalized handcrafted features with the extracted features from the spectrogram. Finally, the merged feature vector will input a fully connected layer to produce the final classification. The output value of the fully connected layer is in the range of 0 and 1 where the value of the final classification label will be decided by:

(5) $$label=\left\{\begin{array}{cc} normal & \mathrm{if}\;x\;<=\;0.5\\ abnormal & \mathrm{if}\;x\;>\;0.5 \end{array}\right.$$

where *x* is the output value of the fully connected layer.

## 3. Experimental Setup and Result Discussion

### 3.1. Dataset Setup

There are 17 different rhythm types in the data sets, as described previously. However, in the experiment, the data were classified according to 1 for abnormal and 0 for normal.

There are 26,532 abnormal ECG rhythms for the rhythm dataset and 1,199,065 normal ECG rhythms. Since there were much more normal rhythm data than anomalous rhythm data, dataset balancing was necessary. The normal rhythm dataset was constructed by randomly selecting 26,532 samples from the original normal rhythm dataset. As a result, the reconstructed dataset has 26,532 abnormal ECG rhythms and 26,532 normal rhythms. For the final dataset, 53,064 ECG rhythms were generated. A total of 90% of the data were used as the training dataset, and 10% were used as the testing dataset. Therefore, the training set contains 47,758 ECG rhythms, and the testing dataset contains 5306 ECG rhythms.

For generating the heartbeat dataset, there is a slight difference. According to Section 2.2, the only abnormal heartbeats that occur in normal rhythm are used for training and testing. There are no Right Bundle Block Beat in these two databases, and Pace Beat occurs in the normal rhythm. Therefore, there were 14 heartbeat types in the training and testing dataset. Similar to the generation of rhythm dataset, the training and testing dataset split follows a 9:1 ratio. Finally, the generated training dataset contains 43,516 heartbeats, and the testing dataset contains 4850 heartbeats. Moreover, the normal and abnormal heartbeats in both datasets are 50–50 split so that the testing result will be nonbiased.

The spectrograms were generated using Short Time Fourier Transform (STFT) described in Section 2.2.3. The dimension of the output RGB spectrograms is 875 × 656 × 3. The spectrogram power values are encoded as an RGB color image to emphasize the difference in one version. The power spectrum power values are simply coded as a greyscale image where an integer represents each pixel in a second version.

### 3.2. Model Training and Testing

A total of 80% data is used as training during the training step, and 20% data is used as validation. The learning rate decay technique is used during the training to determine the best learning rate. The learning rate decay adopts an initially large learning rate and then decays it by a certain factor after predefined epochs. According to You’s study, an initially large learning rate suppresses the memorization of noisy data while decaying the learning rate improves the learning of complex patterns [35]. In the experiment, the start learning rate is 0.1, and the decay rate is 50%. The neural network architectures used in the CNN block are VGG-16, VGG19, ResNet-18 and ResNet-34. The ResNet-50, ResNet-101 and ResNet-150 architectures are not tested here because these two models have too many layers, so the models’ prediction speed will be slow. Therefore, the models will not be able to provide a diagnosis in real-time. Moreover, to show how the feature merging layer could increase the prediction accuracy, regular VGG 16,19 and Residual Neural Network 18 and 34 were trained as well (Figure 9). The results are compared in Table 3.

#### 3.2.1. Multilead Utilization

As described in Section 2.1, the MIT-BIH database and the European ST database are both collected with two-channel ECG devices. Therefore, a DNN model for each lead signal was trained to take advantage of the multilead signal. The models for each lead were all used for the final classification. As a result, the final prediction is calculated using the following equations:

(6) $$Y=Y_{1}+Y_{2}-0.5$$

where $Y_{1}$ is the prediction from lead one model and $Y_{2}$ is the prediction from lead two model. The label is determined using the following conditions:

(7) $$Y=\left\{\begin{array}{cc} 1, & \mathrm{if}\;Y\geq 0.5\\ 0, & \mathrm{otherwise} \end{array}\right.$$

The models could also be used for individual prediction for another dataset with lead-II channel data. Therefore, the models can be used for further learning with other ECG datasets.

#### 3.2.2. Over-Fitting Prevention

**Figure 10:**
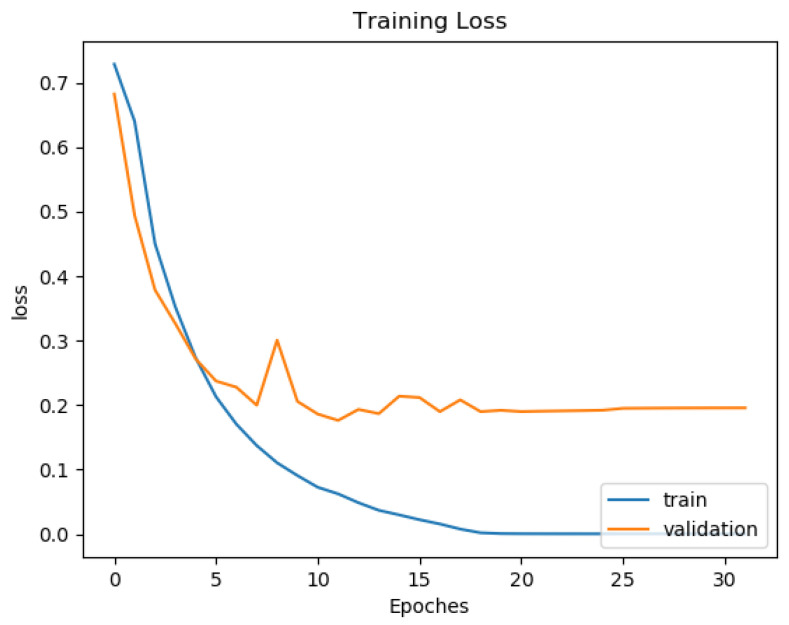
Training and validation loss curve for rhythm classification of ResNet34 Gray.

**Figure 11:**
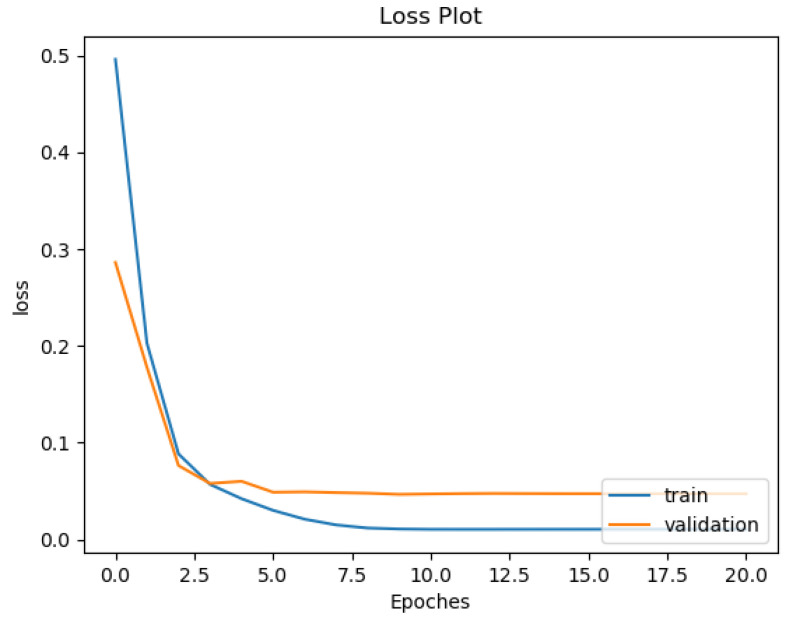
Training and validation loss curve for heartbeat classification of ResNet34 Gray.

Most deep neural network architectures are prone to over-fitting, meaning that if the accuracy of the network on the training set is increased, the accuracy of the network on the actual test set may not improve. This situation indicates that the model is overfitting the training set and cannot generalize all data. During the training of the CNN model, the following two methods were used to prevent over-fitting. The first one consists of adding a dropout layer after the fully connected layer, and the second consists of using an early stopping technique, as shown by [36]. After each training epoch, the model predicts a validation set to determine accuracy. The training process stops if the validation accuracy drops two times in two consecutive epochs. The training and testing were computed on a workstation with Intel Core i5-8400 CPU and Nvidia Geforce GTX 1080 GPU. One can see in Figure 10 and Figure 11 the training and validation of rhythm and heartbeat classification loss function value vs. epochs. It is clear that both training loss and validating loss converge around 20 epochs. The validation loss is lower than the training loss because of that the validation dataset was never trained by the models, it was only used for over-fitting prevention.

### 3.3. Evaluation Metrics

The evaluation metrics used for comparison with ground truth are the following:

- TP: the number of successfully detected abnormal rhythms/heartbeats;
- FP: the number of wrongly detected abnormal rhythms/heartbeats;
- TN: the number of successfully detected normal rhythms/heartbeats;
- FN: the number of wrongly detected normal rhythms/heartbeats;
- Sensitivity (SEN) = TP/(TP + FN);
- False Alarm Rate (FAR)= 1 − Specificity = FP/(FP + TN);
- Positive Predictive Value (PPV) = TP/(TP + FP);
- Accuracy (ACC) = (TP + TN)/(TP + FP + TN + FN).

The main goal is to find the most abnormal rhythms and heartbeats on the ECG signal. The chosen metrics could show whether the algorithm detects abnormal cases. The sensitivity is also called the true positive rate, which shows the detection rate among all abnormal cases. The false alarm shows how many normal cases are detected as abnormal cases. The positive predictive value indicates the number of real abnormal cases among all detected abnormal cases. The accuracy is the overall accuracy for both normal and abnormal detection.

### 3.4. Comparison Results and Discussion

#### 3.4.1. Rhythm Classification Result Discussion

In Table 3, one can compare the result of the proposed method and its comparison to state-of-art algorithms. In the Table, ‘RGB’ indicates that the model was trained with RGB spectrogram, and ‘Gray’ suggests that the model was trained with a grayscale spectrogram. Regular ResNet corresponds to the results obtained by the typical Residual neutral network, shown in Figure 9, that are trained with only the spectrograms. Proposed ResNet corresponds to the new Residual Neural Network architecture, shown in Figure 8. The ‘Time(s)’ column shows how many seconds the models used to predict the test dataset.

Compared to results shown in the Table 3, the proposed models achieved better accuracy and could detect more various rhythm types. Furthermore, the proposed model with feature merging layers performs better than the regular ResNet models. In addition, when comparing the proposed model to Zihlmann’s model [13] which is only trained with a spectrogram, one can see that our approach had much higher accuracy. This demonstrates that prior knowledge, such as handcrafted features, can increase the accuracy of heart rhythm classification.

The accuracy difference between the RGB spectrogram-trained models and Grayscale spectrogram-trained models should be minimal. Since the RGB spectrogram uses different colors to express power density, the grayscale spectrogram expresses the power density using its pixel intensities. However, as the effect for model training, the RGB spectrograms increase the learning capacity of the model since it contains more channels. The result in the Table supports this finding as well. The average accuracy difference between VGG networks is around 3%. The accuracy difference between regular ResNet is around 1%. In addition, the accuracy difference between ResNet with feature merging layer is none. For a regular residual neural network, the models trained with RGB spectrograms have a lower prediction accuracy (about 1%) than those trained with Grayscale spectrograms. This indicates that the model could be overwhelmed by irrelevant information containing three RGB spectrogram channels. However, there is no significant difference between the models trained with RGB spectrogram and those trained with grayscale spectrogram after adding the feature merging layer to the regular residual neural network. This shows that the handcrafted features have a heavier weight than the CNN layer extracted features for modelling the rhythm signal. From the time aspect, the Grayscale spectrogram-trained models took about half the time than the RGB spectrogram-trained models.

For the VGG Neural Network architecture, the models trained with grayscale spectrogram are better than those trained with RGB spectrograms. Additionally, the accuracy for models trained with both spectrogram and handcrafted features is slightly better than those trained with spectrograms only. The VGG19 models have the best overall accuracy compared to the VGG16 models. For the Residual Neural Network architecture, the ResNet18, ResNet34, and ResNet50 have different layers, where the ResNet18 has the least layers, and the ResNet50 has the most layers. The number of layers also indicates the number of trainable variables. More trainable variables mean that the model is harder to learn, or in other words, the model takes a long time to converge. Moreover, during the prediction step, the larger network takes a longer time than the smaller network. The ‘Time’ column in the Table also shows that the ResNet18 models used less time than ResNet34 and ResNet50 models. In addition, the ResNet50 models did not have the best performance in the experiment. The reason could be that more trainable variables in the network caused the model to be easier to overfit to the training dataset than the other models; therefore, it performed worse on the test dataset. By comparing the VGG and ResNet architectures, it is clear that the ResNet architecture performs better than the VGG architecture on accuracy and takes less time for a prediction.

By comparing all the models, the proposed ResNet18-Gray held the highest accuracy with the third-lowest false-alarm rate. The other proposed ResNet-18 and ResNet-34 network models also have similar performances. However, between these models, the proposed ResNet-18 Gray only took 55 s to complete the prediction of the test dataset, which is the fastest model compared to other models. Therefore, the system should produce a high anomaly-detection accuracy and low false-alarm rate in clinical heart anomaly detection. Furthermore, the abnormality detection system should predict as fast as possible; therefore, the proposed ResNet18-Gray model should be the best choice for clinical applications.

#### 3.4.2. Heartbeat Classification Result Comparison

In Table 4, one can compare the result of the proposed method with state-of-art algorithms. The naming of the proposed model is similar to the one described in Section 3.4.1.

Similar to the rhythm classification, three different datasets were tested using both proposed ResNet and regular ResNet. As one can see in Table 4, the proposed ResNet models are better than the standard ResNet models in all metrics. Moreover, the proposed ResNet Models also performed better than Sen’s algorirhm [14] which is only using spectrogram and CNN. Sen’s algorithm achieved 99.57% sensitivity, 2.99% false-alarm rate, 96.72% positive predictive value and 98.21% accuracy detecting three types of heartbeats. The performance metrics were very similar to the regular ResNet-34 model and are 1% less accurate than the proposed ResNet-34 model. This shows that the handcrafted feature could increase the accuracy of ECG heartbeat signal recognition. The overall performance between the models that used the Grayscale spectrogram dataset and the RGB spectrogram dataset is similar for the rhythm classification. The accuracy difference for all metrics was about 1%. For the number of ResNet layers, the 34-layer ResNet models had better performance than the 18-layer ResNet models on the heartbeat datasets. This is caused by the fact that there is less information on the heartbeat spectrogram since there is only one heartbeat. In addition, as shown in Table 4, the ResNet models were faster and more accurate than the VGG models. This result indicates that the residual convolutional neural network architecture is more suitable for processing the ECG STFT spectrogram.

Compared to results found in the literature, the proposed models generally performed better except when compared to Ubeyli’s and Ye’s algorithms. However, Ubeyli’s model [8] has only been tested on 359 heartbeats. Therefore the result may not be suitable for all different heartbeat types. Similar with the Xia’s algorithm [37], their two models could only detect one rhythm; that is, either premature ventricular contraction or supraventricular premature beat. Compared to Ye’s algorithm [12], the performance is quite similar. Ye’s algorithm has 0.18% better overall accuracy, but the proposed model has a better positive predictive value, which means the proposed algorithm could more accurately detect anomalies in the ECG signal. One possible reason for this situation may be caused by Ye’s test data sets containing more normal heartbeats than abnormal heartbeats, so that correct detection of normal heartbeats could increase the overall detection accuracy. Another factor that could affect the model’s accuracy performance is that the training and testing data sets for the proposed model only contain the abnormal heartbeat found in normal rhythms. Therefore, the sample is smaller than the irregular heartbeat on all ECG signals. Still, some rhythm classes are harder to classify, since some heartbeat types are easy to detect but do not exist in normal rhythms, such as paced and Right Bundle block beats.

In conclusion, by comparison, the proposed ResNet34-Gray performs the best at heartbeat classification. It has 98.80% sensitivity, 0.45% false-alarm rate, 99.54% positive prediction value, and 99.18% accuracy. It took 279 s to complete the prediction on the test dataset, which is very fast compared to other models. Compared to the existing state-of-art algorithm, the proposed algorithm has a better predictive value which is higher by 0.9%, and the other three metrics are only lower by between 0.01% and 0.3%. Therefore, the proposed algorithm displays a better performance at detecting ECG signal anomalies.

**Table 4:** Classification result of heartbeats.

Method	A/N	Types	TP	FP	TN	FN	SEN	FAR	PPV	Accuracy	Time(s)
Christov [17]-morphology	18,378/47,239	5	18,042	1604	45,635	336	98.17%	3.40%	91.84%	97.04%	N/A
Christov [17]-frequency	18,378/47,239	5	17,590	1459	45,780	788	95.71%	3.09%	92.34%	96.58%	N/A
Chazal [18]-frequency	4317/34,394	4	4108	1962	32,432	209	95.16%	5.70%	67.68%	94.39%	N/A
Ubeyli [8]	269/90	4	268	2	88	2	99.26%	2.22%	99.26%	99.89%	N/A
Llamedo [38]	5441/44,188	3	4752	2238	41,950	689	87.34%	5.06%	67.98%	94.10%	N/A
Ye [12]	20,745/65,264	16	20,557	286	64,978	188	99.09%	0.44%	98.63%	99.32%	N/A
Zhang [11]	5653/44,011	4	5248	4869	39,142	405	92.84%	11.06%	51.87%	89.38%	N/A
Thomas [9]	26,626/67,268	5	22,900	1300	65,968	3726	86.01%	1.93%	94.63%	94.65%	N/A
Kiranyaz [23]	7366/42,191	5	6539	1228	40,963	827	88.77%	2.97%	84.19%	95.85%	N/A
Rajesh [10]	8000/2000	5	7677	33	1967	323	95.96%	1.65%	99.57%	96.44%	N/A
Sahoo [7]	807/244	4	798	5	239	9	98.88%	2.04%	99.38%	98.67%	N/A
Xia-VEB [37]	300/-	1	-	-	-	-	98.1%	0.1%	99.84%	99.7%	N/A
Xia-SVEB [37]	300/-	1	-	-	-	-	97.2%	0.1%	99.57%	99.8%	N/A
Sen [14]	1154/1305	3	1149	39	1266	5	99.57%	2.99%	96.72%	98.21%	N/A
Regular VGG16-RGB	2425/2425	14	2196	75	2350	229	90.56%	3.09%	96.70%	93.73%	784
Regular VGG19-RGB	2425/2425	14	2171	58	2367	254	89.53%	2.39%	97.40%	93.57%	862
Regular VGG16-Gray	2425/2425	14	2276	45	2380	149	93.86%	1.86%	98.06%	96%	613
Regular VGG19-Gray	2425/2425	14	2359	22	2403	66	97.28%	0.91%	99.08%	98.19%	701
Proposed VGG16-RGB	2425/2425	14	2324	49	2376	101	95.84%	2.02%	97.94%	96.91%	774
Proposed VGG19-RGB	2425/2425	14	2188	57	2368	237	90.23%	2.35%	97.46%	93.94%	861
Proposed VGG16-Gray	2425/2425	14	2366	28	2397	59	97.57%	1.15%	98.83%	98.21%	612
Proposed VGG19-Gray	2425/2425	14	2367	28	2397	58	97.61%	1.15%	98.83%	98.23%	707
Regular ResNet18-RGB	2425/2425	14	2338	53	2372	87	96.41%	2.19%	97.78%	97.11%	388
Regular ResNet34-RGB	2425/2425	14	2354	60	2365	71	97.07%	2.47%	97.51%	97.30%	453
Regular ResNet18-Gray	2425/2425	14	2373	22	2403	52	97.66%	0.91%	98.47%	99.08%	212
Regular ResNet34-Gray	2425/2425	14	2377	19	2406	48	98.02%	0.78%	99.21%	98.62%	272
Proposed ResNet18-RGB	2425/2425	14	2390	26	2399	35	98.56%	1.07%	98.92%	98.74%	381
Proposed ResNet34-RGB	2425/2425	14	2394	32	2393	31	98.72%	1.32%	98.68%	98.70%	444
Proposed ResNet18-Gray	2425/2425	14	2402	18	2407	23	99.05%	0.74%	99.26%	99.15%	209
Proposed ResNet34-Gray	2425/2425	14	2396	11	2414	29	98.80%	0.45%	99.54%	99.18%	279

#### 3.4.3. Discussion and Future Work

Based on the comparison in Section 3.4.1 and Section 3.4.2, The proposed neural network models perform better than other algorithms in Table 3 and Table 4. The novelty of the proposed neural network structure is that it combines the features extracted from the STFT spectrogram using a CNN layer with handcrafted features. Therefore, it utilizes information from both time and frequency domains. In addition, the proposed algorithm can be easily adapted to different ECG databases with variable ECG signal lengths and can detect both abnormal rhythms and abnormal heartbeats from the ECG signal. Most detection algorithms found in the literature are designed only to detect abnormal rhythms or heartbeats.

In clinical practice, the proposed algorithm could help cardiologists to identify areas with structural anomalies on long-term ECG recordings. The program could process the entire recording and provide a report if there are any structural anomalies found. Moreover, the research could be expanded to detect a specific rhythm or heartbeat if the label of the detecting classes is set to 1.

In this research, the interference that affects the ECG signals measurement and anomaly detection is not discussed, since the MIT-BIH database and European ST database had reduced noise. However, high levels of noise could affect the ECG signal measurement and anomaly detection. Based on a previous survey [4], there were two groups of noise on the ECG signal. One is nonphysiological noise, and the other is physiological noise. Equipment problems cause the first one, and the latter is caused by the patient, such as muscle activities, skin interference, and body motion. The physiological noise is much harder to remove because it is unpredictable. In [3], an automatic adaptive noise-removal algorithm was proposed to remove two significant types of physiological noise: baseline wander and motion artifacts. Moreover, there are some target-detection algorithms that could detect anomalies from noisy signals such as [39,40,41]. The research will be expanded to build a wearable ECG device database to collect noise-contaminated ECG signals for future work. By doing so, one can research how noise will affect anomaly detection on ECG signals.

## 4. Conclusions

This paper proposes a new Convolutional Neural Network (CNN) architecture that models the ECG signals using Short Time Fourier Transform (STFT) spectrogram and handcrafted features. The proposed architecture could adapt the latest CNN architectures to process the ECG spectrogram and combine the extracted features with handcrafted features. The proposed algorithm could be used on rhythm and heartbeat classifications with corresponding handcrafted features. The Residual Neural Network (ResNet) architecture is the best architecture for ECG spectrogram. The proposed ResNet contains a feature-merging layer that combines the CNN extract features from spectrograms and handcrafted features. A series of experiments have been conducted to show that these feature-merging layers could improve the ResNet model’s accuracy. According to the experimental result, the new algorithm has superior performance than the current state-of-the-art algorithms. The best model for rhythm classification in the experiment is the proposed ResNet18-Gray. It could classify 17 different rhythm types into normal and abnormal classes with an exceptional accuracy of 99.79%, a sensitivity of 99.74%, a false-alarm rate of 0.15%, and a positive predictive value of 99.85%, which makes this new algorithm the most practical algorithm for rhythm classification in the literature. The heartbeat classification is used only on normal rhythms to detect abnormal heartbeats in the proposed anomaly-detection algorithm. The proposed algorithm is better at detecting state-of-art heartbeat classification algorithms. The best model for heartbeat classification in the experiment is the ResNet34-Gray. It could classify 14 different heartbeat types into normal and abnormal classes with an accuracy of 99.18%, a sensitivity of 98.80%, a false-alarm rate of 0.45%, and 99.54% positive predictive value. The results show that the proposed algorithm has the best detection rate for abnormal heartbeat compared to other algorithms found in the literature. The proposed approach also illustrates that in some situations, the introduction of handcrafted features can significantly improve the performance of neural networks.

## Figures and Tables

**Table 1:** Complete Description of Rhythms Types.

Symbol	Meaning	Database
NSR	Normal sinus rhythm	M, E
AB	Atrial bigeminy	M, E
AFIB	Atrial fibrillation	M, E
AFL	Atrial flutter	M
B	Ventricular bigeminy	M, E
BII	2${}^{\circ }$ heart block	M
B3	3${}^{\circ }$ heart block	E
IVR	Idioventricular rhythm	M
NOD	Nodal (A-V junctional) rhythm	M
P	Paced rhythm	M
PREX	Pre-excitation (WPW)	M
SAB	Sino-atrial block	E
SBR	Sinus bradycardia	M, E
SVTA	Supraventricular tachyarrhythmia	M, E
T	Ventricular trigeminy	M, E
VFL	Ventricular flutter	M
VT	Ventricular tachycardia	M, E

**Table 2:** Complete Description of Heartbeat Types.

Symbol	Meaning	Database
N	Normal	M, E
LBBB	Left bundle branch block beat	M
RBBB	Right bundle branch block beat	M
PAC	Atrial premature beat	M
a	Aberrated atrial premature beat	M, E
J	Nodal (junctional) premature beat	M, E
S	Supraventricular premature beat	M, E
PVC	Premature ventricular contraction	M, E
F	Fusion of ventricular and normal beat	M, E
e	Atrial escape beat	M
j	Nodal (junctional) escape beat	M, E
E	Ventricular escape beat	M, E
P	Paced beat	M
f	Fusion of paced and normal beat	M, E
Q	Unclassified beat	M

**Table 3:** Classification results of rhythms.

Method	A/N	Types	TP	FP	TN	FN	SEN	FAR	PPV	ACC	Time(s)
AR modeling [5]	713/143	6	706	10	133	7	99.02%	6.99%	98.60%	98.01%	N/A
Acharya Net A [22]	20,807/902	4	19,160	62	840	1647	92.08%	6.87%	99.68%	92.13%	N/A
Acharya Net B [22]	8322/361	4	7946	376	294	67	99.16%	56.12%	95.48%	94.90%	N/A
Zihlmann [13]	-	4	-	-	-	-	-	-	-	82.3%	N/A
Regular VGG16-RGB	2653/2653	17	2522	185	2468	131	95.06%	6.97%	93.17%	94.04%	433
Regular VGG19-RGB	2653/2653	17	2436	235	2418	217	91.82%	8.86%	91.20%	91.48%	452
Regular VGG16-Gray	2653/2653	17	2554	215	2438	99	96.27%	8.10%	92.24%	94.08%	332
Regular VGG19-Gray	2653/2653	17	2544	116	2537	109	95.89%	4.37%	95.64%	95.76%	349
Regular ResNet18-RGB	2653/2653	17	2536	121	2532	117	95.59%	4.56%	95.45%	95.51%	117
Regular ResNet34-RGB	2653/2653	17	2550	131	2522	103	96.12%	4.94%	95.11%	95.59%	152
Regular ResNet18-Gray	2653/2653	17	2599	155	2498	54	97.96%	5.84%	94.37%	96.06%	61
Regular ResNet34-Gray	2653/2653	17	2635	150	2503	18	99.32%	5.65%	94.61%	96.83%	96
Proposed VGG16-RGB	2653/2653	17	2433	173	2480	220	91.71%	6.52%	93.36%	92.59%	425
Proposed VGG19-RGB	2653/2653	17	2476	199	2454	177	93.33%	7.50%	92.56%	92.91%	446
Proposed VGG16-Gray	2653/2653	17	2567	172	2481	86	96.76%	6.48%	93.72%	95.14%	319
Proposed VGG19-Gray	2653/2653	17	2572	134	2519	81	96.95%	5.05%	95.05%	95.95%	345
Proposed ResNet18-RGB	2653/2653	17	2648	15	2638	5	99.81%	0.57%	99.44%	99.62%	110
Proposed ResNet34-RGB	2653/2653	17	2646	10	2643	7	99.74%	0.38%	99.62%	99.68%	144
Proposed ResNet18-Gray	2653/2653	17	2646	4	2649	7	99.74%	0.15%	99.85%	99.79%	55
Proposed ResNet34-Gray	2653/2653	17	2649	8	2645	4	99.85%	0.30%	99.70%	99.77%	90

## Data Availability

Data used in this research is not provided.
